# Comparative Transcriptomic Immune Responses of Mullet (*Mugil cephalus*) Infected by Planktonic and Biofilm *Lactococcus Garvieae*

**DOI:** 10.3389/fcimb.2022.887921

**Published:** 2022-05-23

**Authors:** Feng-Jie Su, Thirunavukkarasu Periyasamy, Meei-Mei Chen

**Affiliations:** ^1^Department of Veterinary Medicine, National Taiwan University, Taipei, Taiwan; ^2^Department of Biotechnology, Nehru Arts and Science College, Coimbatore, India

**Keywords:** immune system, Lactococcus garvieae, biofilm, planktonic, fish

## Abstract

*Lactococcus garvieae* is an important pathogen of fish, associated with high rates of mortality and infection recurrence in summer or stressful conditions. Chronic infection and disease recurrence have also been reported to be associated with biofilms. However, the impact of biofilm and planktonic bacterial infection on fish immune responses remains unclear. In this study, *de novo* sequencing was used to compare differences of the spleen transcriptome in planktonic- and biofilm-infected mullets. Among the 181,024 unigenes obtained, 3,392 unigenes were associated with immune response genes. Comparative analysis of the gene expression between infection with the *L. garvieae* planktonic type and biofilm type identified a total of 3,120 and 3,489 differentially expressed genes in response to planktonic and biofilm infection, respectively, of which 1,366 and 1,458 genes were upregulated, and 1,754 and 1,458 genes were downregulated, respectively. Gene ontology enrichment analysis of immune genes identified genes involved in the complement system, toll-like receptor signaling, and antigen processing, which were further verified by qPCR. Additionally, genes encoding TLR2, IL-1β, TNF-α, C7, and MHC class II peptides were downregulated in response to biofilm infection. Importantly, the results show that biofilm infection induces a different immune pathway response compared with planktonic bacterial infection and, furthermore, illustrates that the prevention of biofilm formation may be a necessary and new strategy for controlling bacterial infection in aquaculture.

## Introduction

*Lactococcus garvieae* is a common fish pathogen that not only causes many deaths among infected fish, but also tends to be transmitted by carriers after infection, which continues the spread of the pathogen. Infected fish present with bleeding, ascites, meningitis, and other symptoms (Chen et al., 2002; Vendrell et al., 2006; Evans et al., 2009). *L. garvieae* outbreaks have also been found on many farms, with mortality rates as high as 75%, untreatable with antibiotics (Eldar et al., 1999; Shahin et al., 2021). The current study finds that the biofilm produced by *L. garvieae* prevents antibiotics from killing the bacteria (Dahdouh et al., 2020). This may lead to recurrence and persistence of host streptococcosis.

A bacterial biofilm is a community of microorganisms that attaches to the surface of a substance or tissue and contains bacteria in a complex matrix (Flemming and Wingender, 2010). Biofilm formation involves multiple stages, starting with the reversible attachment of planktonic bacteria to the surface. Single colonies can produce a polymer matrix formation biofilm. The establishment of mature biofilms can release single bacterial planktonic cells that are able to adhere to other sites (Garrett et al., 2008). In humans, biofilm bacteria can spread to multiple body surfaces, including cardiac, pulmonary, and epithelial surfaces (Hernández-Jiménez et al., 2013), and they can cause fibrous cysts in the respiratory tract (Bjarnsholt et al., 2009).

There are many bacteria that form biofilms, which are resilient to adverse environmental conditions and resistant to antibiotics and host immune system attacks (Stewart and Costerton, 2001). Biofilms also characteristically lead to difficulties in controlling complications related to infection, thereby limiting treatment options. Additionally, bacterial biofilms protect bacteria from external damage, and they can escape host immune responses by promoting persistent chronic infections, which are characterized by tissue fibrosis (Costerton, 2001).

Transcriptome analysis is a powerful tool capable of providing valuable information about immunity during infection. Several studies have reported similar infections by bacteria, including *L. garvieae*, in a number of fish (Byadgi et al., 2016) Similarly, iridescent viruses infect many species of fish, including grouper (Chen et al., 2012; Zhang et al., 2013; Tran et al., 2015; Zhu et al., 2015; Wang et al., 2016). These studies have provided useful information and the basis for follow-up research. Given that many reports have found that immune transmission pathways, such as TLR and JAK-STAT, are activated in infected fish, it is possible to identify relevant immune factors that oppose bacterial infection. However, there is currently a lack of information on the effect of biofilms on the expression of immune-related genes in infected fish.

Thus, this study aimed to investigate the spleen transcriptome in planktonic- and biofilm-infected mullets. We first confirmed characteristics of *L. garvieae* biofilm, including antibiotic resistance and the type of suspension, using scanning electron microscopy (SEM). We then collected the spleens of infected fish and analyzed changes of immune-related genes to understand the impact of biofilm formation on infected fish. We also collected information at different time points to understand differences in the immune impact on plankton- and biofilm-based infection in fish, and we found that biofilm infection induces a different immune pathway compared with planktonic infection in mullets.

## Materials and Methods

### *L. garvieae* Biofilm or Planktonic Culture and Quantification

The *L. garvieae was* isolated from a disease outbreak in a mullet farm in Taiwan. TSA agar with 5% sheep blood (OxoidTW, Creative Media Plate, New Taipei City, Taiwan) was used to isolate *L. garvieae* colonies, which were identified by 16S rRNA PCR (Zlotkin et al., 1998). The *L. garvieae* biofilm culture and quantification method was modified from the procedure used for a previous study (Su and Chen, 2021). Single colonies were culture in 3 ml of BHI medium in 6-well plates in static incubation for 48 hours. Thereafter cell scrapers were used to collect the biofilm, the biofilm was washed with phosphate-buffered saline (PBS), centrifuged at 6,000 rpm for 30 minutes, and then the precipitated biofilm bacteria were collected. Dimethylmethylene Blue Assay (DMMB) was used to quantify and coat the plate to calculate CFU/ml. The biofilm was quantified using a method described elsewhere (Tote et al., 2008). For the preparation of the *L. garvieae* planktonic suspension, single colonies were cultured in 30 ml of (Brain Heart Infusion) BHI medium and rotated at 100 rpm at 28°C for 7 hours. Then the pellet was washed with PBS, centrifuged at 10000 rpm for 30 minutes, and the planktonic bacteria were collected. Optical densityOD was used to quantify and coat the plate to calculate the CFU/ml value. The biofilm and planktonic suspensions were collected on microscope slides and observed using SEM.

### Experimental Fish

Sixty-six mullet (*Mugil cephalus*) (weight 14 ± 1.02g)(body length 15.2 ± 0.2 cm) were purchased from an aquaculture farm in Hsinchu, Taiwan. The fish culture system is referenced in previous study (Su and Chen, 2021). The spleens of five fish were randomly selected for TSA agar separation and PCR confirmation of *L. garvieae* infection. In case of any bacterial colonies present on cultured plates and PCR identify *L. garvieae* signal, all experimental fish will be excluded from experiment.This study was approved by the Animal Care Use Committee of the National Taiwan University (protocol no. B201800003).

### Biofilm and Planktonic Bacteria Challenge and Total RNA Extraction

The planktonic and biofilm bacteria challenge was modified from a previous study (Byadgi et al., 2016; Levipan et al., 2018). The planktonic group (plank) included six fish that were anesthetized and injected intraperitoneally with 1 × 10^7^ CFU of bacteria per fish. The bacteria were diluted in PBS. The biofilm group (biofilm) included six fish that were anesthetized and injected intraperitoneally with 1 × 10^7^ CFU of bacteria per fish. The bacteria were diluted in PBS. The control group included six fish that received only PBS. All fish were anesthetized with 30ppm tricaine mesylate (MS-222). Samples were taken 24 hours after infection for RNA sequencing.

To investigating immune genes at different time points, 48 healthy mullet fish were randomly divided into three groups. The plank group included 16 fish, which were anesthetized, injected intraperitoneally with 1×10^7^ CFU of bacteria, and suspended in 100 μL of PBS. In the biofilm group, 16 fish were anesthetized, each fish was injected intraperitoneally with 1×10^7^ CFU of bacteria, and suspended in 100 μL of PBS. The 16 fish in the control group received PBS only. After that, four fish in each group each underwent splenectomy and then underwent RNA extraction at 6, 12, 24, and 48 hours. Additionally, four fish in each group each received a spleen and then underwent RNA extraction, with some tissue plated on blood agar to confirm bacterial infection at 6, 12, 24, and 48 hours. We extracted total RNA using an RNA kit (Geneaid Co., Ltd., New Taipei City, Taiwan).

### Library, Sequencing, Transcriptome Assembly, Gene Functional Annotation

The library preparation was performed using a modified version of a procedure described elsewhere (Jie et al., 2019). A total of 2 µg of RNA per sample was used for RNA sequencing. mRNA was transcribed to DNA using the ToolsQuant RT Kit (BIOTOOLS, New Taipei City, Taiwan). The RNA sequencing library was sequenced using the Illumina NovaSeq 6000 platform (Novogene Co., Ltd., New Taipei City, Taiwan), and paired-end reads were generated. Raw data were recorded in a FASTQ file containing sequence information (reads) and corresponding sequencing quality information. For all samples, post-filtered clean reads were selected using Trinity software to complete the transcriptome reconstruction process (Grabherr et al., 2011). Raw RNA sequencing data were submitted to NCBI SRA database (accession numbers SAMN26139190 [biofilm group] and SAMN26139191 [plank group]). Gene function was annotated based on the following databases: Nr (NCBI non-redundant protein sequences), Nt (NCBI non-redundant nucleotide sequences), Pfam (protein family), KOG/COG (Clusters of Orthologous Groups of proteins), Swiss-Prot (a manually annotated and reviewed protein sequence database), KO (KEGG Ortholog database), GO (Gene Ontology).

### Differentially Expressed Genes (DEGs) Analysis

We used edgeR software to identify and analyze differential gene expression between the plank and biofilm groups. We used q-values instead of p-values according to the methods described for a previous study (Shannon et al., 2003). Q value<0.005 & |log2 (foldchange)|>1 was set as the threshold for indicating a significantly differential expression. We analyzed GO and KEGG enrichment differential unigens with reference to previous research (Kanehisa et al., 2007; Young et al., 2010).

### Real-Time PCR

The cDNA was synthesized from 1 μg of total RNA using the GoScript™ Reverse Transcriptase Kit (Promega Co., Ltd,New Taipei, Taiwan). Quantitative real-time PCR was conducted using the primers listed in **Table 1**. Real-time PCR was amplified using an ABI Stepone Plus Real-Time PCR machine (Applied Biosystems, Waltham,MA, USA) with TOOLS SYBR Green qPCR Mix (BIOTOOLS Co., Ltd,New Taipei Taiwan) following the manufacturer’s instructions. The threshold cycle (Ct) values were obtained from each sample. Relative gene expression levels were evaluated using the −ΔΔCT method.

**Table 1:** Primer design.

Name	Sequence	Tm (°C)	Reference
IL-1β-F	GAGGAGCTTGGTGCAGAACA	61.4	(Byadgi et al., 2016)
IL-1β-R	CTTTGTTCGTCACCTCCTCCA		
C3-F	GCATCACGCTCCTTGTCTTT	61.4	(Byadgi et al., 2016)
C3-R	ACCACTATGCCACAAGAACATC		
β-actin-F	TGCAGTCAACATCTGGAATC	59	(Byadgi et al., 2016)
β-actin-R	ATTTTTGGCGCTTGACTCAG		
TNF-α-F	GCGCAGTCTGTCATTGGTT	60	(Byadgi et al., 2016)
TNF-α-R	ACTGGACACGCTCACTGTAGTG		
*MHC I-F*	GCAGAACCAGAGGCTTCAACA	59	(Byadgi et al., 2016)
*MHC I-R*	TCAGGAGGAGTTGTGTCTATGAAC		
*IL-8-F*	CACTGCTGGTCGTCCTCATT	59	(Byadgi et al., 2016)
*IL-8-R*	CAGTCGGAGGTCGGAAGTCT		
TLR 2-F	CTTTCTCCTCGTCCCTCTG	59	(Su and Chen, 2021)
TLR 2-R	CGTGTTTGTTGTGGTCT		
*MHC II-F*	TCTGGCCTTCCTCTTGTAGT	59	in this paper
*MHC II-R*	GCCAGCCTGAAAGAACCT		
*C7-F*	CTGCCCTCAATGTAAATTTCCT	59	in this paper
*C7-R*	TTTGAGAACCTGGGAGCCT		
*IL-10-F*	GTGTTGCGCTTCTACGTT	59	in this paper
*IL-10-R*	AGCAGAGAGATTAGCGTGAAT		

### Statistical Analysis

All data were subjected to ANOVA followed by Duncan’s multiple range tests. A *p*-value <0.05 was considered significant.

## Results

### Comparing DMMB Quantification and Observed SEM Characterization of Plankton and Biofilm Types in *L. garvieae*

**Figure 1:**
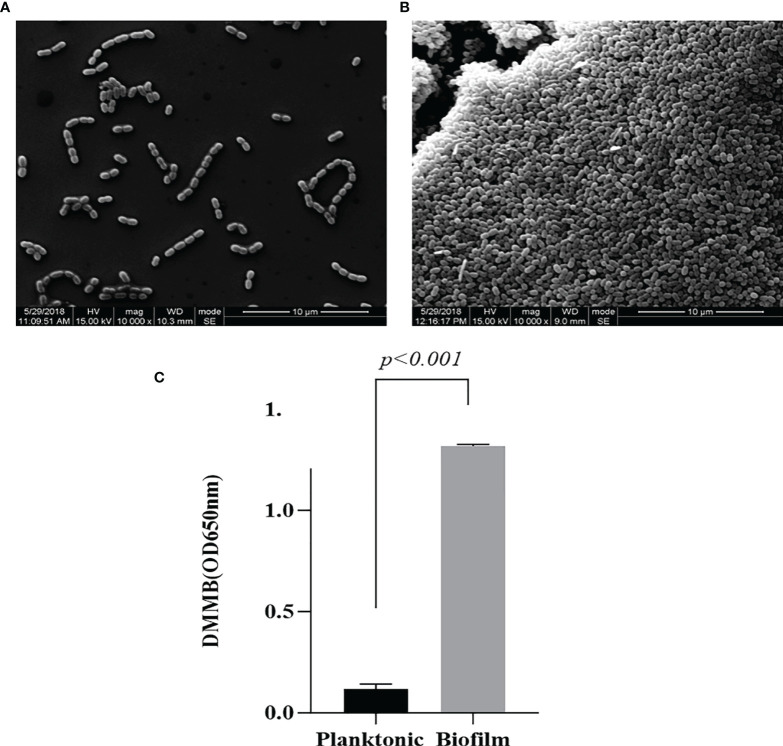
Difference between *Lactococcus garvieae* planktonic type and biofilm type. **(A)** *L. garvieae* planktonic type. **(B)** *L. garvieae* biofilm type. **(C)** The mean values of *L. garvieae* planktonic type and biofilm type DMMB under the same 10^7^ CFU/ml. Mean *p*-values were calculated by one-way ANOVA (*p* < 0.001).

Observation of *L. garvieae* planktonic (**Figure 1A**) type and biofilm (**Figure 1B**) types by SEM revelaed that when the *L. garvieae* biofilm forms, it aggregates to form a three-dimensional structure and protects the interiorly situated bacteria. DMMB staining was used to observe the characteristics of bacterial biofilm and planktonic type under the same 10^7^ CFU/ml bacterial concentration. The mean DMMB values were 0.118 and 1.319 for the planktonic type and biofilm type, respectively (**Figure 1C**). These findings revealed that there is a significant difference between planktonic type and biofilm type.

### Planktonic Group and Biofilm Group Transcriptome Sequencing and Assembly in Mullet Spleens

The total numbers of raw reads obtained from the spleens of fish from the control (PBS), planktonic-infected, and biofilm-infected groups were 49,554,708, 48,990,144, and 46,165,134, respectively. After removing reads with adaptors and low-quality reads from the original data, the numbers of clean reads for each group were 47,909,728, 45,930,138, and 43,611,838, respectively. The numbers of clean bases, which were determined by the numbers of clean reads multiplied by read length (G), of the control (PBS), planktonic, and biofilm groups were 7.2 G, 6.9 G, and 6.5 G, respectively (**Table 2**).

**Table 2:** RNA-seq data production.

Sample	Raw reads	Clean reads	Clean bases	GC Content (%)
PBS	49554708	47909728	7.2G	50.03
plank	48990144	45930138	6.9G	49.82
biofilm	46165134	43611838	6.5G	49.88

**Figure 2:**
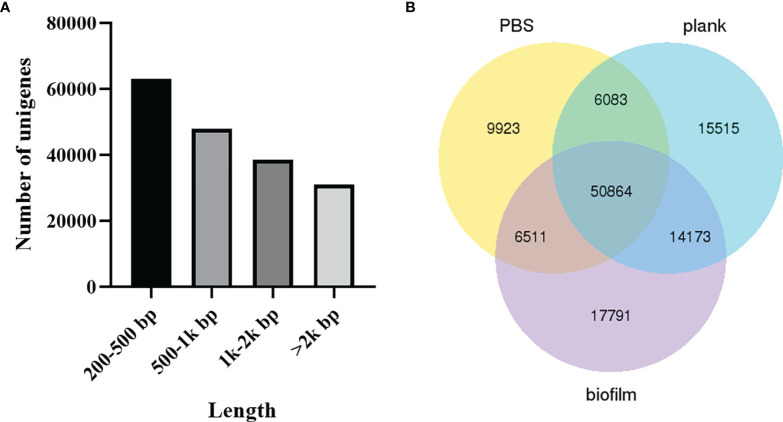
The length distribution of unigenes and contigs. **(A)** *Lactococcus garvieae* planktonic- and biofilm-infected mullet unigene length distribution. The x-axis indicates the number of unigenes, and the y-axis indicates the length size (bp). **(B)** Venn diagram of PBS, plank, and biofilm intergroup expression.

The unigene range was from 200 to 31,113. There were 63,196 (34.91%) unigenes with 200-500 bp, 48,026 (26.53%) unigenes with 500-1k bp, 38,689 (21.37%) unigenes with 1k-2k bp, and 31,113 (17.19%) unigenes with >2k bp (**Figure 2A**). The Venn diagram shows the number of genes in each group, and the overlapping areas show the number of genes expressed in two or more groups. Totals of 73,381, 78,973, and 81,249 contigs were detected for the PBS, plank, and biofilm groups. There PBS, plank, and biofilm groups had 50,864 contigs in common (**Figure 2B**). Comprehensive gene function annotation of unigenes using seven databases (Nr, Nt, Pfam, KOG, Swiss-Prot, KEGG, GO) is shown in **Table 3**.

**Table 3:** The annotated gene number of unigenes and percentage.

Database	Number of unigenes	Percentage (%)
Annotated in NR	90587	50.04
Annotated in NT	107099	59.16
Annotated in KO	43806	24.19
Annotated in SwissProt	76703	42.37
Annotated in PFAM	71762	39.64
Annotated in GO	71797	39.66
Annotated in KOG	43035	23.77
Annotated in all Databases	24291	13.41
Annotated in at least one Database	121393	67.05
Total Unigenes	181024	100

**Figure 3:**
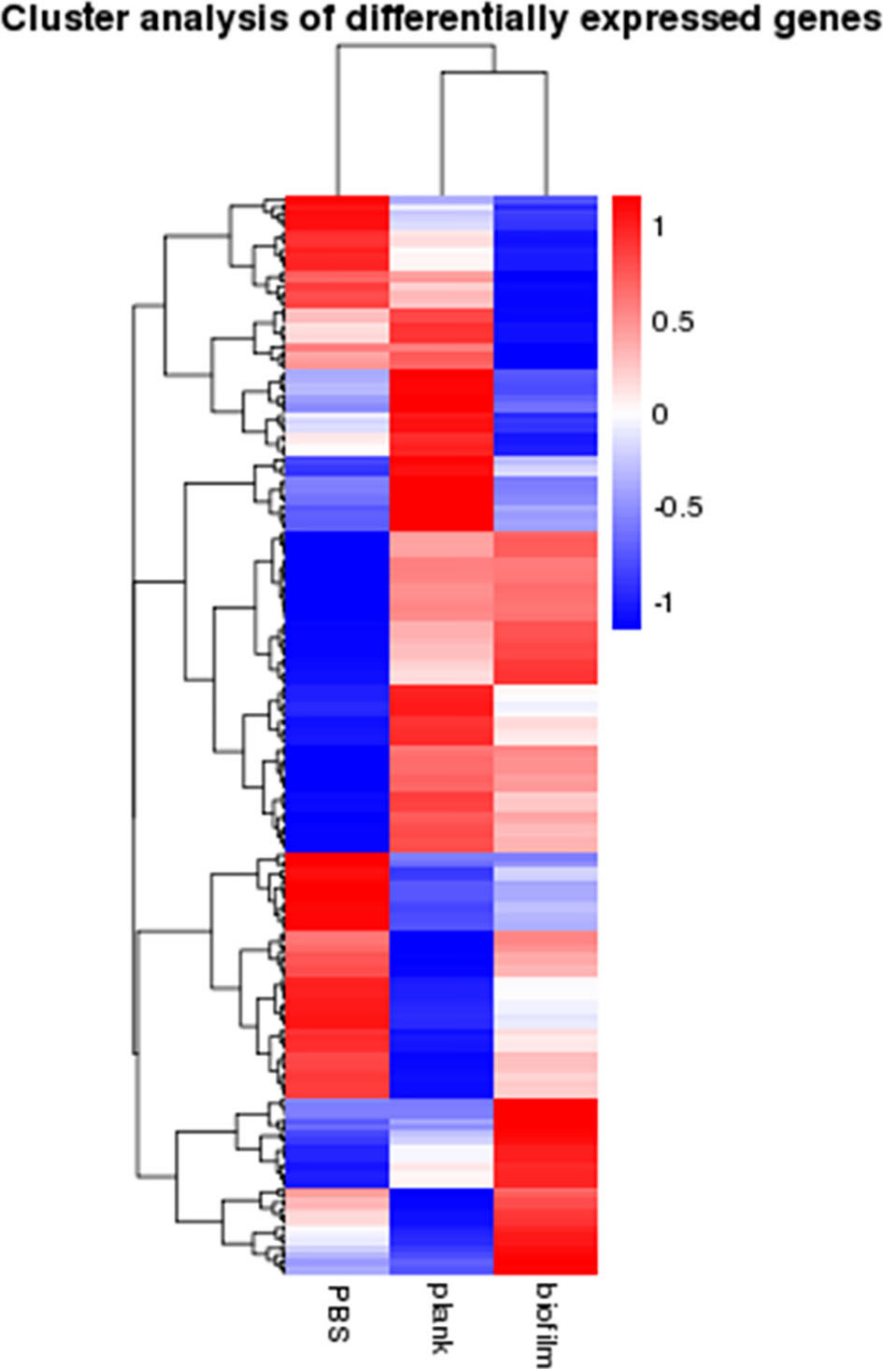
Cluster analysis of gene expression differences. The heatmap analysis of gene expression differences between the PBS, plank, and biofilm groups. Weak and strong correlations between variables are shown in green and red, respectively.

Signature genes with previously unknown functions can be identified *via* clustering analysis to find genes expression patterns. **Figure 3** shows a heat map of mRNAs expressed as genes—the planktonic and biofilm groups yielded different trend from the PBS group.

### Planktonic Group and Biofilm Group GO Classification of DEGs

**Figure 4:**
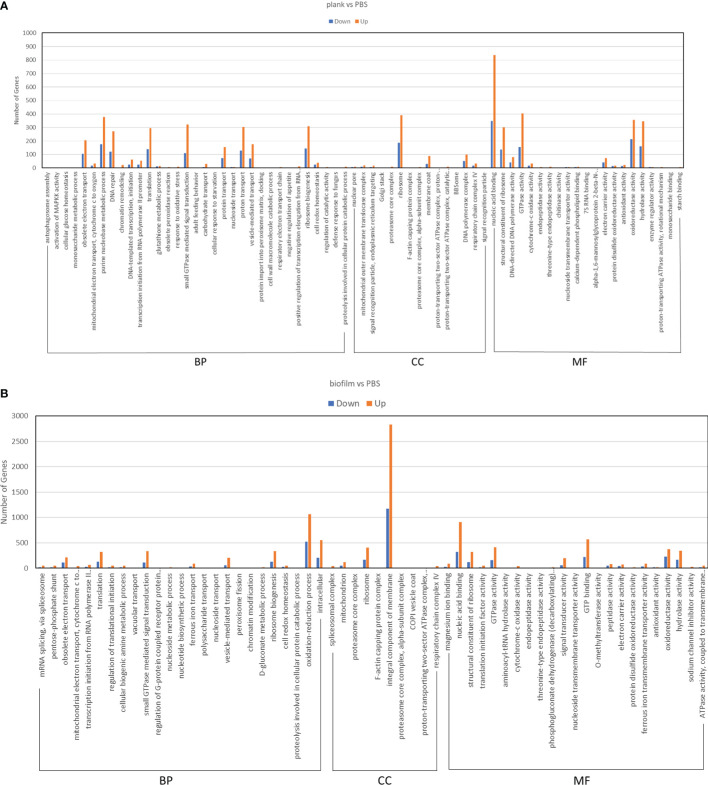
GO enrichment analysis of DEGs in *Lactococcus garvieae* planktonic- and biofilm-infected mullets. GO analysis was divided into three categories: biological process (BP), cellular component (CC), and molecular function (MF). **(A)** plank group; **(B)** biofilm group.

To understand DEG function, we also mapped all discovered DEGs in terms of the GO database. The plank group was found to classify 69 groups in the GO analysis (**Figure 4A**). The biofilm group was found to classify 58 groups in the GO analysis (**Figure 4B**). In the group infected by planktonic bacteria, a total of 2,695 genes were downregulated, and 5,971 were upregulated. In the biofilm-infected group, we found that 4,438 genes were downregulated, and 10,700 were upregulated. GO analysis was divided into three categories: biological process (BP), cellular component (CC), and molecular function (MF). The GO analyses for the plank and biofilm groups also revealed that related genes, in terms of BP, were dominant with PBS group.

### Immune-Related Responses to Planktonic and Biofilm Infection

At 24 hours post–planktonic infection, gene expression of TLR2 and TLR13 were upregulated, whereas expression of TLR2 and Complement component 7 was downregulated in the biofilm-infected group. The planktonic and biofilm group were mapped to KEGG with the Toll-like receptor (TLR) signaling pathway and Complement and Coagulation cascades. (**Supplementary Figures 1, 2**). In terms of interferon regulatory factors, the biofilm group yielded less activity than the plank group. The biofilm group yielded more expression of tissue fibrosis factors, such as cystic fibrosis transmembrane conductance regulator and arginase, than the plank group (**Table 4**).

**Table 4:** Immune immune-related responses to *Lactococcus garvieae* planktonic and biofilm infection in mullet spleens.

	Fold change –plank/PBS	Fold change –biofilm/PBS
Arginase	2.19	4.63
Complement component 7	2.28	-2.41
Cystic fibrosis transmembrane conductance regulator	-1.51	1.2
Heat shock protein 70	1.74	5.38
Interferon gamma	5.76	4.93
Interferon gamma receptor 2	2.95	1.71
Interferon regulatory factor 1	3.70	2.47
Interferon regulatory factor 4	2.53	1.77
Interferon regulatory factor 5	2.85	1.40
Interferon regulatory factor 9	2.23	1.87
Interferon-induced GTP-binding protein Mx1	2.71	2.77
Interleukin 1 beta	1.34	-1.17
Interleukin 10	1.38	5.25
Interleukin 8	4.85	2.07
Major histocompatibility complex, class I	4.01	1.91
Major histocompatibility complex, class II	2.73	-1.76
Nuclear factor of activated T-cells, cytoplasmic 2	7.71	3.93
Signal transducer and activator of transcription 1	2.18	1.55
Signal transducer and activator of transcription 3	3.88	1.49
Signal transducer and activator of transcription 5B	2.05	2.68
Signal transducer and activator of transcription 6	3.51	1.41
T-cell receptor beta chain V region	1.81	-1.82
TLR13	-3.71	3.15
TLR2	2.02	-2.12
TLR7	2.80	1.89
TRAF family member-associated NF-kappa-B activator	2.81	-3.07
Interleukin-5 receptor	-1.11	1.10

### Validation of RNA-seq Data by qRT-PCR

**Figure 5:**
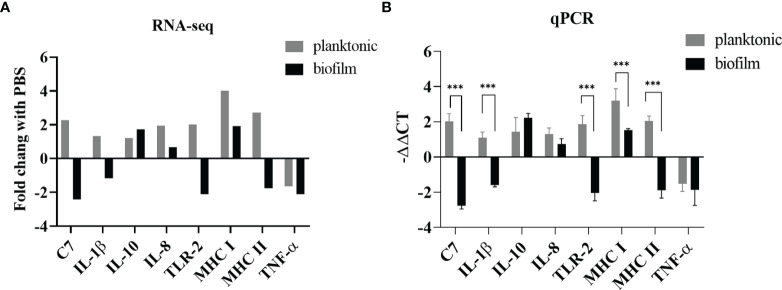
Comparison of gene expression determined by RNA-seq and qPCR. **(A)** The relative expression levels from the RNA-seq analysis were compared with the PBS group. **(B)** The relative expression levels from the qPCR analysis were compared with the PBS group. *p*-values were calculated by two-way ANOVA (*p* < 0.01 **, *p* < 0.001 ***). Each value is the mean of six samples. Bars represent mean ± standard deviation.

In this study, we designed primers with reference to some previous articles on *L. garvieae* published by Byadgi et al. (Byadgi et al., 2016). Of these eight genes, those expressing C7, IL-1β, TLR2, MHC-II, and TNF-α were downregulated in the biofilm-infected group, whereas those expressing MHC-I, IL-10,IL-8 were upregulated in both the planktonic and biofilm groups. As shown in **Figure 5**, we found that the expression of all five of these genes in both groups exhibited concordance in RNA-seq (**Figure 5A**) and qRT-PCR (**Figure 5B**) analysis.

### The *L. garvieae* Biofilm Causing Immune Downregulation in Mullets

**Figure 6:**
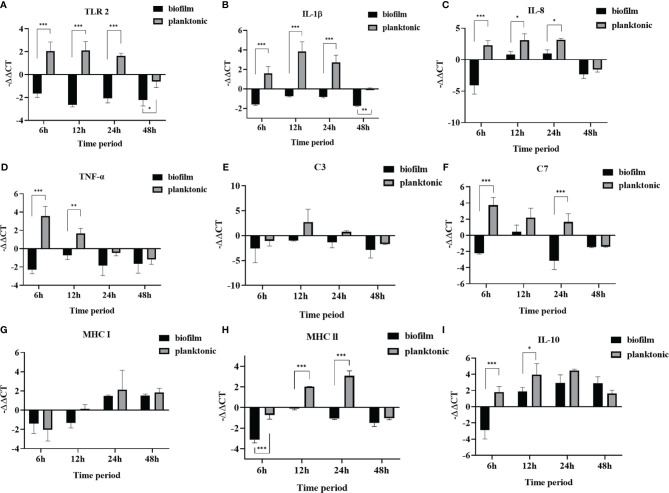
The relative expression levels of immune genes in mullet spleens after infection with planktonic and biofilm *Lactococcus garvieae*. **(A)** *TLR 2*, **(B)** *IL-1β*, **(C)** *IL-8*, **(D)** *TNF-α*, **(E)** *C3*, **(F)** *C7*, **(G)** *MHC I*, **(H)** *MHC II*, **(I)** *IL-10*. Data are presented as mean ± standard deviation. *p*-values were calculated by two-way ANOVA (*p*<0.05*, *p* < 0.01**, *p* < 0.001***).

The results showed that *L. garvieae* colonies could be isolated at 6, 12, 24, and 48 hours after infection with planktonic and biofilm bacteria (**Table 5**).For the *TLR2, IL-1β* gene, we found that it was upregulated at 6, 12, and 24 hours and downregulated at 50 hours after infection in the planktonic group. However, in the biofilm group, it was downregulated at 6, 12, 24, 48 hours after infection (**Figures 6A, B**). The *IL-8* gene was upregulated at 6, 12, and 24 hours and downregulated at 50 hours after infection in the planktonic group; in the biofilm group, the *IL-8* was upregulated at 12 and 24 hours after infection and downregulated at 6 and 48 hours after infection (**Figure 6C**). The *TNF-α* gene was upregulated at 6 and 12 hours and downregulated at 24 and 48 hours after infection in the planktonic group; in the biofilm group, it was downregulated at 6, 12, 24, and 48 hours after infection (**Figure 6D**). Expression of the *C3* complement gene was downregulated at 6 and 48 hours and upregulated at 12 and 24 hours after infection in the planktonic-infected group, whereas it was consistently downregulated after infection in the biofilm-infected group (**Figure 6E**). The *C7* complement gene was upregulated at 6, 12, and 24 hours and downregulated at 48 hour after infection in the planktonic group (**Figure 6F**). The *MHC I* gene was initially downregulated at 6 hours after infection, but it was upregulated at 12, 24 and 48 hours in the planktonic group; in the biofilm group, the *MHC I* gene was upregulated at 6 and 12 hours after infection but downregulated at 24 and 48 hours (**Figure 6G**). The *MHC II* gene was upregulated at 12 and 24 hours after infection in the planktonic group but downregulated at 6 and 48 hours. In the biofilm group, the *MHC II* gene was downregulated at 6, 12, 24, and 48 hours after infection (**Figure 6H**). The *IL-10* gene was upregulated at 6, 12, 24, and 48 hours after infection in the planktonic group; in the biofilm group, the *IL-10* gene was downregulated at 6 hours after infection but upregulated at 12, 24, and 48 hours after infection (**Figure 6I**). Additionally, these findings also revealed that the trend for the planktonic group was similar to that of the biofilm group at 48 hours after infection.

**Table 5:** Isolation of *Lactococcus garvieae* from mullet spleens on TSA agar. Confirmation of *L. garvieae* using 16S rRNA PCR.

	6 h	12 h	24 h	48 h
Planktonic group	+/+/+/+	+/+/+/+	+/+/+/+	+/+/+/+
Biofilm group	+/+/+/+	+/+/+/+	+/+/+/+	+/+/+/+
PBS group	‐/‐/‐/‐	‐/‐/‐/‐	‐/‐/‐/‐	‐/‐/‐/‐

## Discussion

*L. garvieae* has always presented a challenge in the aquaculture industry of Taiwan, but the adoption of high-density fish farming methods has led to significant transmission of disease and even repeated recurrences of infection. Recurrence and persistent infections have mainly been caused by bacterial biofilm, and the extracellular matrix of bacterial biofilm can cause immunosuppression, drug resistance, and anti-inflammation (Rasmussen and Givskov, 2006; Chen and Wen, 2011; Taff et al., 2013). Biofilm is mostly an aggregate rich in environmental DNA, extracellular polysaccharides, and other substances (Flemming and Wingender, 2010). It was previously reported that *Streptococcus pyogenes* produces biofilms and accumulates in injured tissues, making it difficult to recover from infection; it also elicits no inflammatory response (Neely et al., 2002).

Furthermore, it has been reported that the biofilm of the fish pathogen *Flavobacterium columnare* can adhere to the surface of an object within 6 hours and form biofilm colonies within 24 hours (Cai et al., 2013). Isiaku et al. found that biofilms may affect a host’s inflammatory response and persist in the body for a long time (Gu et al., 2019). Similarly, our findings also showed that fish from the planktonic-infected group exhibited the same immunosuppressive signals as those from the biofilm-infected group at 48 hours. In contrast, the biofilm-forming ability of stained bacteria is different. Currently, the most common way to determine biofilm strength is *via* DMMB assay (Xu et al., 2016). From 2006 to 2019, we successively isolated 33 strains of *L. garvieae* with different biofilm strengths. We determined that bacterial strain No. 930330 had the strongest biofilm-producing ability, and using a combination of SEM and transcriptome analysis, we used this strain to examine differences between biofilm and planktonic bacteria as well as compare the impact of biofilm and planktonic *L. garvieae* on mullet immune mechanisms. Although an analysis of the transcription factors related to mullet infection has been previously published (Byadgi et al., 2016), the findings of the present study not only showed similar results with planktonic bacterial infections, but also enabled a comparison between the spleen gene expression patterns of fish infected by either planktonic or biofilm *L. garvieae*. We assembled a total of 181,024 unigenes that were 77.84%, 76.38%, and 76.23% mapped in the PBS, planktonic, and biofilm groups, respectively. We then used these data were to analyze several common index factors and compare immune response differences between biofilm and planktonic infection.

The complement system is an important immunological mechanism for immune protection in fish. The complement components of fish have pro-inflammatory roles akin to those of mammals (Grayfer et al., 2018). However, as the complement system of fish has a mild mammalian-like pro-inflammatory effect, it protects fish from an initial infection more effectively than its mammalian counterpart (Holland and Lambris, 2002). C3 has always played an important role in the three pathways of the complement response. When C3 comes into contact with various microbial surfaces, a series of subsequent assembly events results in their destruction (Muller-Eberhard, 1986). Further, it has been shown that trout C3a, C4a, and C5a have a chemical attraction to kidney phagocytes and peripheral blood lymphocytes in the head, and they enhance phagocytosis of kidney leukocytes (Li et al., 2004). We found that the C3 response gradually increased after planktonic *L. garvieae* infection, a finding which is in line with those of a 2016 study (Byadgi et al., 2016). However, we also found that it was downregulated 48 hours after infection, a difference which may be related to biofilm formation. In contrast, C3 and C7 expression was consistently downregulated at all examined time points in the biofilm-infected group, which suggests that biofilms lead to decreased complement capacity in infected fish and is similar to a previous report that found biofilm formation in *S. pneumopresent* was an efficient means of evading both the classical and alternative complement pathways of the host immune system (Domenech et al., 2013). Based on our findings, we conclude that, although the complement system is activated, it is unable to effectively overcome *L. garvieae* infection. Therefore, a future research direction that warrants additional study is to identify the substances or factors produced by *L. garvieae* biofilms that inhibit the complement system.

TLRs are involved in the regulation of many innate immune factors in mammals. (Kumar et al., 2009). The characteristics and signaling of fish TLRs share a high degree of structural similarity with mammalian TLR systems. However, fish TLRs also have many unique features and exhibit considerable diversity, possibly due to their different evolutionary histories and environments (Palti, 2011). In acute infection, TLRs can distinguish the type of pathogen and play an important role in coordinating appropriate adaptive immune responses (Takeda et al., 2003). Teleost fish are considered to have a primitive immune system, and there is considerable scientific interest to compare their innate immunity and adaptive defence mechanisms with those found in mammals. In the past few decades, 16 types of TLRs have been found for bony fish (Palti, 2011), and currently, the genomic sequences of the TLR protein family have been completed for zebrafish (*Danio rerio*) and pufferfish (*Takifugu rubripes*) (Oshiumi et al., 2003; Jault et al., 2004; Meijer et al., 2004).

As a member of the TLR family of proteins, TLR2 is characterized by an extracellular domain containing 18 to 20 tandem leucine repeats, a transmembrane domain, and a cytoplasmic TOLL/IL-1 receptor domain for signal transduction (5). It has been demonstrated that TLR2 directly participates in the recognition of pathogen-associated molecular patterns (PAMPs) and activates pro-inflammatory cytokines and type I interferons through a conservative single pathway, which can be MyD88 dependent, MyD88 independent, or TRIF independent. TLR2 is a conservative component of gram-positive bacteria recognized by acidic receptors, such as lipoteichoic acid, peptide derivatives, lipoproteins, yeast reproduction, protozoan parasites, and lipopolysaccharides of gram-negative bacteria (Aliprantis et al., 1999; Underhill et al., 1999; Takeda and Akira, 2005). Experimentally, we found that planktonic *L. garvieae* caused upregulation of TLR2, but expression decreased to levels similar to that found in the biofilm-infected group at 48 hours. Results from the biofilm group showed that TLR2 was downregulated at 6 hours and persisted through 48 hours. Prior studies have similarly reported that planktonic infection causes upregulation of TLR2 (Basu et al., 2012; Samanta et al., 2012; Zhang et al., 2014; Byadgi et al., 2016); however, expression changes after subsequent biofilm formation have not usually been examined. And we can find that group A found the downstream genes of TLR channels, such as IL-1β, TNF-α, and IL-8 at 48 hours, which are closely related to the trend of the biofilm group. We believe that this is caused by the formation of biofilm in planktonic bacteria *in vivo*.

As another immune mechanism against pathogens, T-cell receptor (TCR) signalling is achieved by T-cell antigens through the antigen ligand presented by the MHC on the recognized antigen-presenting cell (Svensson et al., 1997). Unlike the MHC class II (MHC II) system that is not found in all fish, MHC class I (MHC I) molecules play an important role in the adaptive immune performance of fish, and in fact, the MHC I system may replace the function of MHC II to confer the ability to resist pathogens (Basta and Alatery, 2007; Wilson, 2017). In the present study, we found that planktonic infection affected the TCR signaling pathway and activated both MHC I and MHC II, and that our findings are consistent with the experimental results of Byadgi et al. (2016). These findings are of particular interest because, although biofilm infection suppressed gene expression in other immune systems, MHC I was upregulated after 24 hours, indicating that the MHC I system warrants further investigation towards managing biofilm-related infection in fish.

In conclusion, this study provides evidence of the immune gene downregulation of bacterial biofilms in fish and shows that downregulation occurs within 48 hours after infection by planktonic bacteria. Further, our findings may help answer questions about *L. garvieae* in aquaculture, as this pathogenic bacterium continues to pose challenges in controlling disease outbreaks and infections in fish. Therefore, controlling the interactions between biofilms and hosts may be used as a sound future strategy for the prevention and control of pathogenic bacteria in aquaculture.

## Data Availability Statement

The datasets presented in this study can be found in online repositories. The names of the repository/repositories and accession number(s) can be found in the article/**Supplementary Material**.

## Ethics Statement

The animal study was reviewed and approved by Animal Care Use Committee of the National Taiwan University.

## Author Contributions

F-JS contributed to the development of the study methods, investigation, data curation, formal analysis, and data analysis. M-MC and TP thoroughly edited the manuscript. All authors have read and agreed to the published version of the manuscript.

## Conflict of Interest

The authors declare that the research was conducted in the absence of any commercial or financial relationships that could be construed as a potential conflict of interest.

## Publisher’s Note

All claims expressed in this article are solely those of the authors and do not necessarily represent those of their affiliated organizations, or those of the publisher, the editors and the reviewers. Any product that may be evaluated in this article, or claim that may be made by its manufacturer, is not guaranteed or endorsed by the publisher.
