# Supplementary material for: Comparative Transcriptomic Immune Responses of Mullet (Mugil cephalus) Infected by Planktonic and Biofilm Lactococcus Garvieae
Source: Front Cell Infect Microbiol. 2022 May 23;12:887921. doi: 10.3389/fcimb.2022.887921 (PMC9168659; doi:10.3389/fcimb.2022.887921)
Supplement: Supplementary file 1 [file Image_1.pdf]

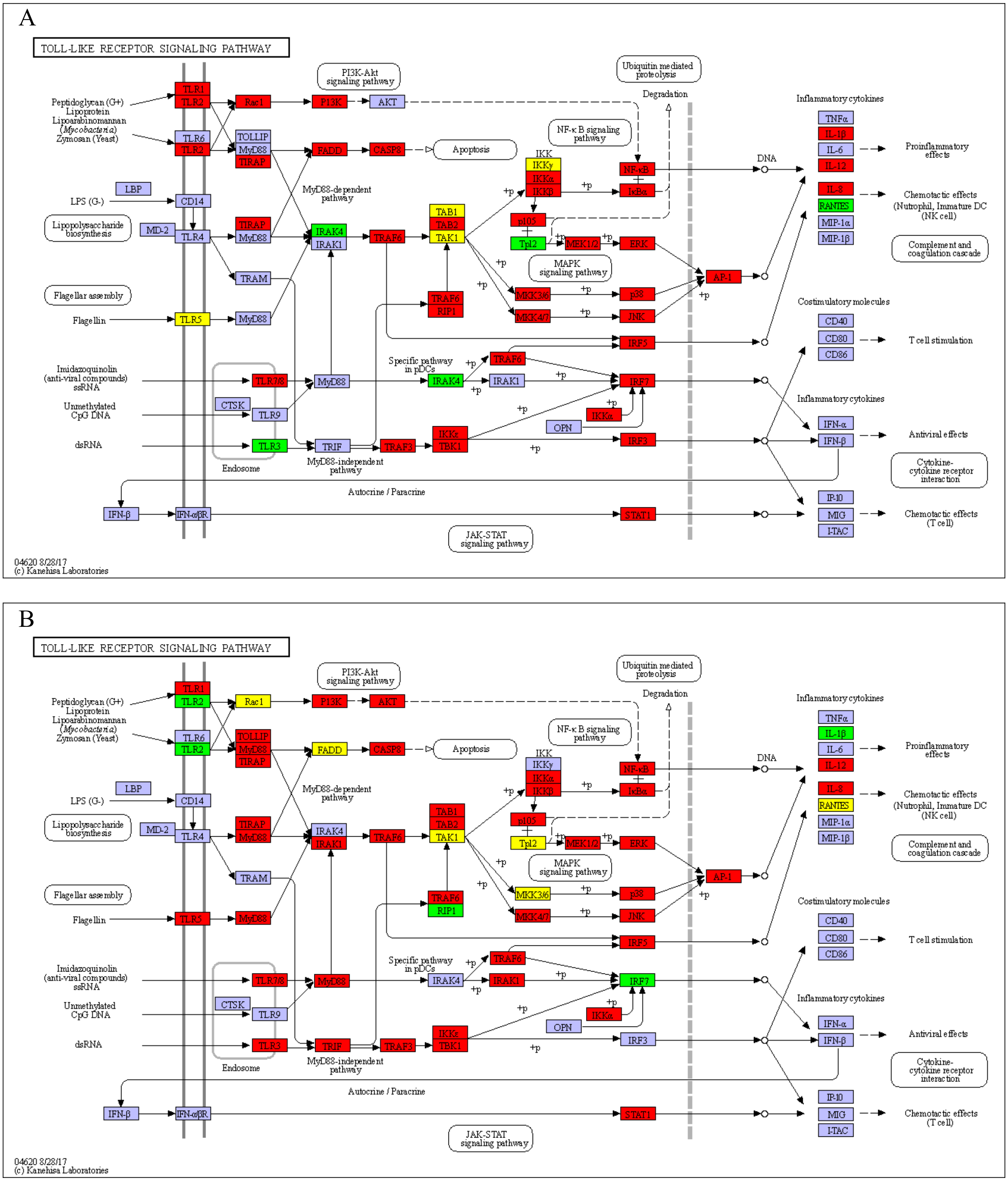

Supplementary Figure 1. TLR signalling pathway mapped by KEGG. (A) plank group. (B)biofilm group. Red indicates upregulated expression. Green indicates downregulated expression. Yellow indicate unchanged expression in transcriptomic profile.

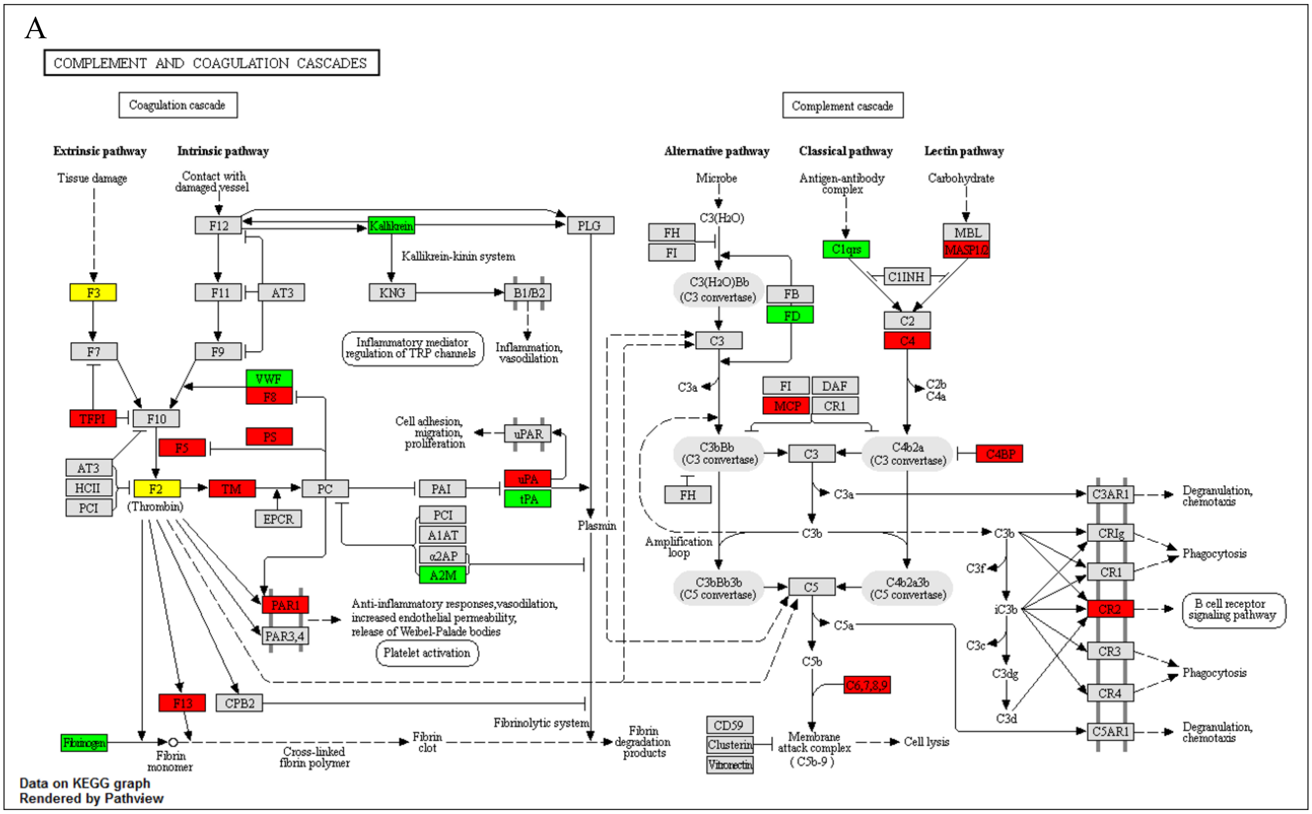
A
COMPLEMENT AND COAGULATION CASCADES
Coagulation cascade
Complement cascade
Extrinsic pathway
Intrinsic pathway
Alternative pathway
Classical pathway
Lectin pathway
Tissue damage
Contact with damaged vessel
Microbe
Antigen-antibody complex
Carbohydrate
F12
Kallikrein
PLG
Kallikrein-kinin system
F3
F11
AT3
KNG
B1/B2
F7
F9
Inflammatory mediator regulation of TRP channels
Inflammation, vasodilation
VWF
F8
TFPI
F10
F5
PS
Cell adhesion, migration, proliferation
uPAR
HCII
PCI
F2
(Thrombin)
TM
PC
PAI
uPA
tPA
EPCR
A1AT
α2AP
A2M
Plasmin
PAR1
PAR3,4
Anti-inflammatory responses,vasodilation, increased endothelial permeability, release of Weibel-Palade bodies
Platelet activation
F13
CPB2
Fibrinolytic system
Fibrinogen
Fibrin monomer
Cross-linked fibrin polymer
Fibrin clot
Fibrin degradation products
C3(H2O)
FH
FI
C3(H2O)Bb (C3 convertase)
FB
FD
C1qrs
C1INH
MBL
MASP1/2
C2
C4
C3
C3a
C2b
C4a
DAF
MCP
CR1
C4BP
C3bBb (C3 convertase)
C4b2a (C3 convertase)
Amplification loop
C3b
C3AR1
Degranulation, chemotaxis
CRIg
Phagocytosis
C3f
iC3b
CR2
B cell receptor signaling pathway
C3c
CR3
C3dg
C3d
CR4
C3bBb3b (C5 convertase)
C5
C4b2a3b (C5 convertase)
C5a
C5b
C6,7,8,9
CD59
Clusterin
Vitronectin
Membrane attack complex (C5b-9)
Cell lysis
C5AR1
Data on KEGG graph
Rendered by Pathview

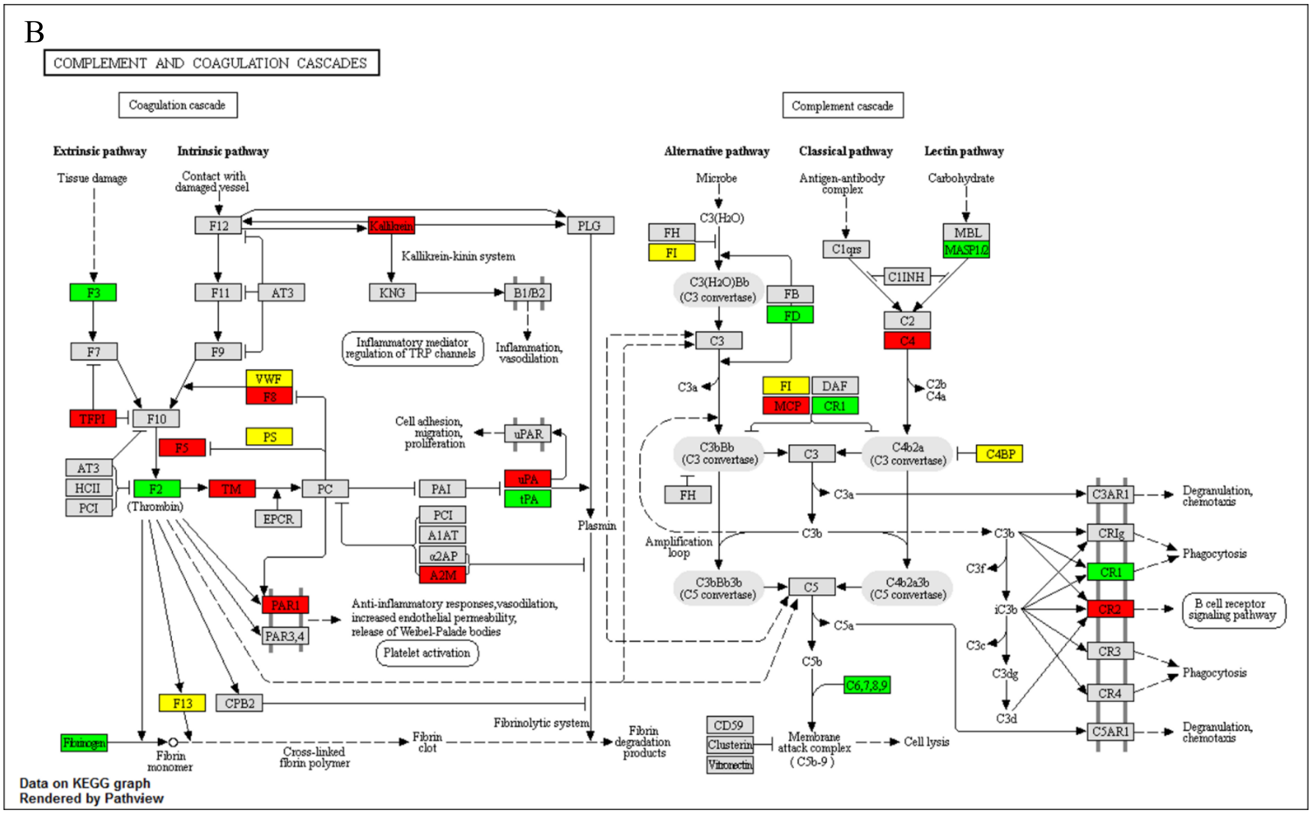
B
COMPLEMENT AND COAGULATION CASCADES
Coagulation cascade
Complement cascade
Extrinsic pathway
Intrinsic pathway
Alternative pathway
Classical pathway
Lectin pathway
Tissue damage
Contact with damaged vessel
Microbe
Antigen-antibody complex
Carbohydrate
F12
Kallikrein
PLG
Kallikrein-kinin system
F3
F11
AT3
KNG
B1/B2
F7
F9
Inflammatory mediator regulation of TRP channels
Inflammation, vasodilation
VWF
F8
TFPI
F10
F5
PS
Cell adhesion, migration, proliferation
uPAR
HCII
PCI
F2
(Thrombin)
TM
PC
PAI
uPA
tPA
EPCR
A1AT
α2AP
A2M
Plasmin
PAR1
PAR3,4
Anti-inflammatory responses,vasodilation, increased endothelial permeability, release of Weibel-Palade bodies
Platelet activation
F13
CPB2
Fibrinolytic system
Fibrinogen
Fibrin monomer
Cross-linked fibrin polymer
Fibrin clot
Fibrin degradation products
C3(H2O)
FH
FI
C3(H2O)Bb (C3 convertase)
FB
FD
C1qrs
C1INH
MBL
MASP1/2
C2
C4
C3
C3a
C2b
C4a
DAF
MCP
CR1
C4BP
C3bBb (C3 convertase)
C4b2a (C3 convertase)
Amplification loop
C3b
C3AR1
Degranulation, chemotaxis
CRIg
Phagocytosis
C3f
iC3b
CR2
B cell receptor signaling pathway
C3c
CR3
C3dg
C3d
CR4
C3bBb3b (C5 convertase)
C5
C4b2a3b (C5 convertase)
C5a
C5b
C6,7,8,9
CD59
Clusterin
Vitronectin
Membrane attack complex (C5b-9)
Cell lysis
C5AR1
Data on KEGG graph
Rendered by Pathview

Supplementary Figure 2. Complement and Coagulation cascades mapped by KEGG. (A) plank group. (B)biofilm group. Red indicates upregulated expression. Green indicates downregulated expression. Yellow indicate unchanged expression in transcriptomic profile
